# A functional SUMO-motif in the active site of PIM1 promotes its degradation via RNF4, and stimulates protein kinase activity

**DOI:** 10.1038/s41598-017-03775-w

**Published:** 2017-06-15

**Authors:** R. Sumanth Iyer, Lynsey Chatham, Roger Sleigh, David W. Meek

**Affiliations:** 1 Division of Cancer Research, Jacqui Wood Cancer Centre, Ninewells Hospital and Medical School, University of Dundee, James Arrott Drive, Dundee, DD1 9SY UK; 2 CXR Biosciences, 2 James Lindsay Place, Dundee Technopole, Dundee, DD1 5JJ UK

## Abstract

The PIM1 serine/threonine protein kinase mediates growth factor and survival signalling, and cooperates potently with c-MYC during tumorigenesis. PIM1 is overexpressed in many human cancers and is a promising target for drug development. PIM1 levels are regulated mainly through cytokine-induced transcription and protein degradation, but mechanisms regulating its activity and levels remain largely unexplored. Here, we show that PIM1 is modified *in vitro* and in cultured cells by the Small ubiquitin-like modifier (SUMO) on two independent sites: K169, within a consensus SUMOylation motif (IK^169^DE^171^) in the active site of PIM1, and also at a second promiscuous site. Alanine substitution of E171 (within the consensus motif) abolished SUMOylation, significantly increased the half-life of PIM1, and markedly reduced its ubiquitylation. Mechanistically, SUMOylation promoted ubiquitin-mediated degradation of PIM1 via recruitment of the SUMO-targeted ubiquitin ligase, RNF4. Additionally, SUMOylated PIM1 showed enhanced protein kinase activity *in vitro*. Interestingly, the E171A mutant was active *in vitro* but displayed altered substrate specificity in cultured cells, consistent with the idea that SUMOylation may govern PIM1 substrate specificity under certain contexts. Taken together, these data demonstrate that the protein kinase activity and levels of PIM1 can be regulated by a covalent post-translational modification.

## Introduction

The proto-oncogene *PIM1* (Proviral Integration site for MuLV site-1) encodes a serine/threonine protein kinase that phosphorylates a wide range of substrates involved in the regulation of important cellular processes such as cell cycle progression, protein translation, metabolism and survival^1^. PIM1 levels are elevated in a significant proportion of haematological malignancies such as lymphomas and solid tumours such as prostate cancer, where its expression correlates with high-tumour grade^2, 3^. More recently, PIM1 was shown to be upregulated in triple-negative breast cancers where it is thought to engage potently in driving c-MYC-dependent transcription^4, 5^. PIM kinases have been shown to mediate therapeutic resistance through various mechanisms and in a range of cellular contexts relevant to human cancer development^6^. PIM inhibitors are therefore considered to be promising targets for cancer therapy and have been a significant focus of drug development.

Unlike most protein kinases, PIM kinases are constitutively active, lack regulatory domains, and do not require any external phosphorylation events for activation. At the protein level, the half-life of PIM1 varies between 5 to 15 minutes in primary cells^7^ and is found to be increased up to 100 minutes in certain tumour cells such as K562, and BV173 expressing the BCR-ABL fusion protein, potentially through HSP90-mediated protection from ubiquitylation and subsequent proteasomal degradation^8, 9^. Other authors have also reported that PIM1 can be destabilized by the protein phosphatase PP2A^10, 11^. Apart from these events, however, there have been no other regulatory post-translational modifications reported for PIM kinases. Furthermore, the pathway(s) by which PIM1 is targeted for ubiquitylation and degradation is incompletely understood and remains a subject of ongoing investigation. A clearer understanding of these events may provide insight into how PIM1 could be efficiently targeted for degradation in cancer therapy.

The Small Ubiquitin-like Modifier (SUMO) is a protein tag that is covalently and reversibly attached to a lysine residue on its protein substrates, in a manner similar to the ubiquitin pathway. SUMOylation regulates important biological processes including gene expression, intracellular transport, chromosome segregation, DNA repair, and protein turnover/stability^12–15^.

In the present study we have explored the mechanisms of regulation of PIM1 in greater depth. We show that PIM1 is covalently modified by SUMO1 and SUMO2/3 in cultured cells on at least two lysine residues, one consensus (i.e. within a SUMOylation recognition motif) and one non-consensus. We find that SUMO modification is a key event in the rapid turnover of PIM1, thereby providing mechanistic detail for the normally short-lived nature of this oncogenic protein kinase. We also show that SUMOylation can directly stimulate the protein kinase activity of PIM1 towards established physiological substrates, demonstrating, for the first time, a non-auto-catalytic mechanism of regulation for this protein kinase. These novel findings offer new insights into PIM regulation and reveal potential opportunities for further therapeutic targeting.

## Results

### PIM1 is modified *in vitro* and in cultured cells by SUMO

To explore whether PIM1 might be regulated post-translationally, potential sites of post-translational modification present in all three PIM kinases (PIM1, 2 and 3) were investigated using a bioinformatics approach. This analysis revealed that K169 of PIM1, K165 of PIM2 and K172 of PIM3 lie within a classic SUMO consensus motif, ψKxE/D, where ψ (psi) is a hydrophobic residue (usually I/V/L/F/M), K is the modified lysine and x is any amino acid^16^.

**Figure 1:**
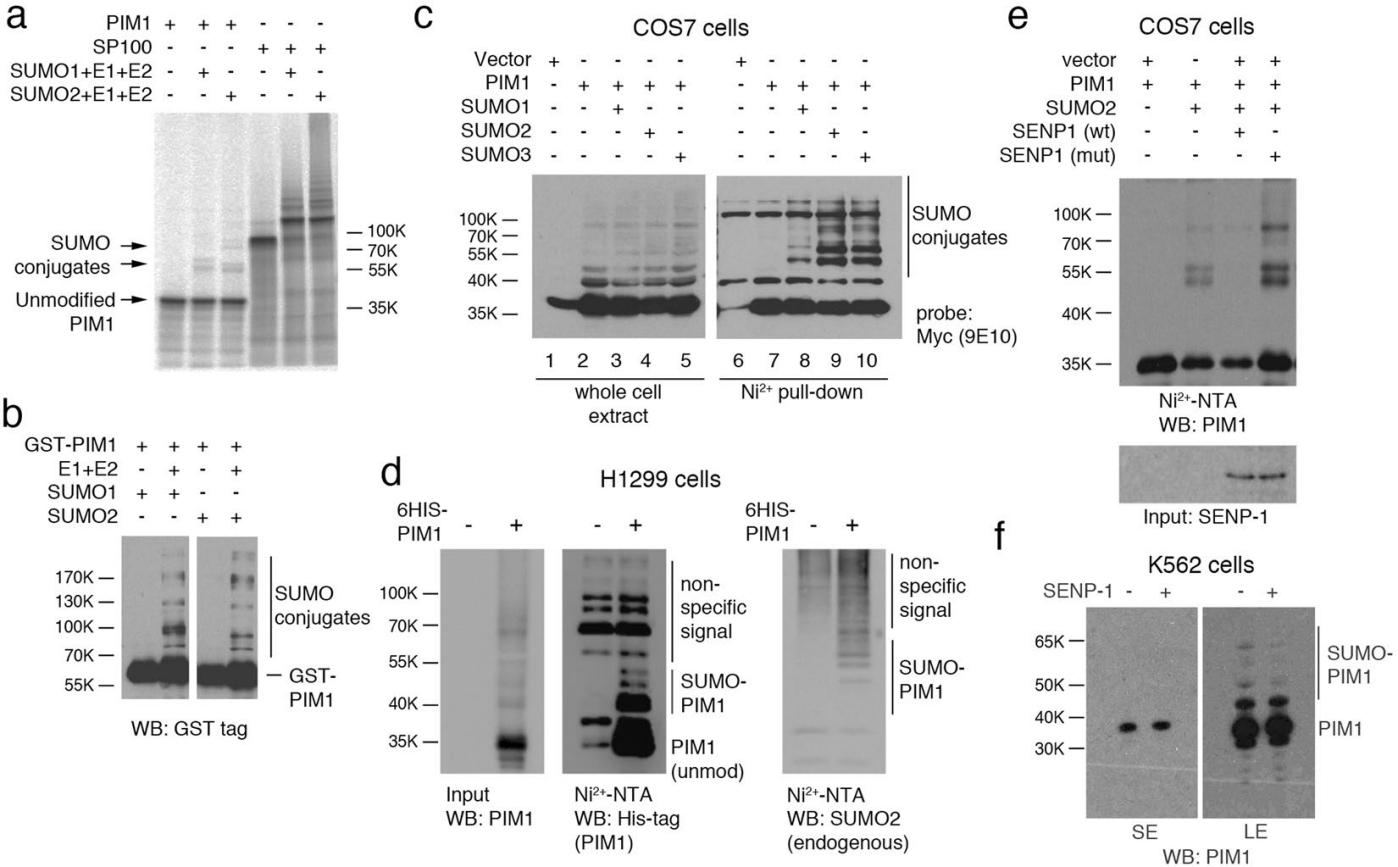
SUMOylation of PIM1 *in vitro* and in cultured cells. (**a**) *In vitro* transcribed and translated ^35^S-methionine labeled PIM1 was incubated with recombinant SAE1/2, UBC9 with SUMO1 or SUMO2 in the presence of ATP-regeneration system. SP100 was used as a positive control in the SUMOylation reaction. SUMOylation of radiolabelled PIM1 was visualized on a Phosphorimager. (**b**) Bacterially expressed and purified GST-PIM1 was incubated in the presence of ATP, recombinant SAE1/2, UBC9 with SUMO1 (left) or SUMO2 (right). SUMOylated PIM1 was detected by western blotting using a GST-tag antibody. (**c**) COS7 cells were transfected with plasmids encoding MYC-tagged PIM1 alone or in combination with 6His-SUMO1, 2 or 3 and SUMOylation assay was carried out using Ni^2+^-NTA beads, 42–48 hours post transfection. PIM1 SUMOylation was analyzed by western blotting of Ni^2+^-NTA pull-down samples using MYC-tag (9E10) antibody. Total levels of PIM1 expressed under each transfection condition were analyzed by western blotting of Input samples using MYC-tag (9E10) antibody. (**d**) H1299 cells were transfected with a plasmid expressing 6His-PIM1, and PIM1 was affinity purified under denaturing conditions as done previously for 6His-SUMO protein. Eluted proteins were analyzed by western blotting using PIM1 (12H8) and SUMO2 antibody to detect SUMOylated PIM1. (**e**) COS7 cells were transfected with plasmids expressing PIM1 alone, or with 6His-SUMO2 in combination with catalytically active Flag-SENP1 (WT) or inactive Flag-SENP1 (MT), and SUMOylation assay was performed to isolate SUMOylated proteins. PIM1 SUMOylation was analyzed by western blotting using PIM1 (12H8) antibody. Western blotting of whole cell lysate or input was done using Flag-tag antibody to confirm expression of SENP1. Empty vector was included, where appropriate, to maintain equal amounts of transfected plasmid DNA. (**f**) Lysates from K562 cells (treated with 20 μM MG132 for 6 hours) were incubated at 30 °C for 30 minutes in the absence or presence of 50 nM recombinant catalytic domain of SUMO protease, SENP1, followed by western blotting using PIM1 (12H8) antibody.

To determine whether PIM1 could be post-translationally modified by SUMO, ^35^S-labelled PIM1 was incubated *in vitro* with the SUMO E1 and E2 enzymes SAE1/2 and UBC9 respectively, in the absence and presence of SUMO1 or SUMO2. Analysis of the products by SDS-PAGE and autoradiography showed the appearance of slower migrating PIM1 species with apparent molecular weights (around 55 kDa) consistent with SUMOylated forms of PIM1 (Fig. 1a). PIM1 showed weak SUMO modification in this experiment compared with SP100 (positive control) suggesting that PIM1 may require an E3 SUMO ligase for efficient modification. Higher molecular weight bands were also observed when purified GST-PIM1 was incubated with SUMO1 or SUMO2 in the presence of the SUMO E1 and E2 enzymes *in vitro* (Fig. 1b).

To determine whether SUMOylation of PIM1 can occur within a cellular context, SUMOylation assays were performed in COS7 cells transfected with plasmids expressing MYC-tagged PIM1 together with plasmids expressing 6His-tagged SUMO1, 2 or 3 (Fig. 1c). While PIM1 was not modified in the absence of ectopically expressed SUMO proteins, SUMO conjugates of PIM1 were detected when either SUMO1, 2, or 3 were co-expressed. Notably, higher molecular weight PIM1-SUMO conjugates run as a doublet around 55 kDa (also observed in Fig. 1a) suggesting that SUMO modification may occur on at least two independent sites. Affinity purification of 6His-tagged PIM1 alone under denaturing conditions in H1299 cells was sufficient to observe SUMOylation by endogenous SUMO2 cells, indicating that (a) it is a substrate for endogenous SUMOylation enzymes, and (b) SUMOylation of PIM1 is not a cell line-specific phenomenon (Fig. 1d). Furthermore, expression of the WT SENP1, but not an inactive SENP1 mutant, led to disappearance of the higher molecular weight PIM1 bands, confirming that the doublet band observed was indeed SUMO-modified PIM1 (Fig. 1e).

To assess whether PIM1 is endogenously SUMOylated, extracts from K562 cells (which express relatively high levels of PIM1) were analyzed by western blotting following treatment, or mock treatment, with the SUMO protease, SENP1 *in vitro*. As shown in Fig. 1f (long exposure), the levels of higher molecular weight bands corresponding to SUMO-modified forms were reduced in the presence of SENP1 with no detectable changes in the levels of the unmodified kinase (short exposure, Fig. 1f). This observation supports the idea that PIM1 SUMOylation occurs endogenously in untransfected cells.

### PIM1 is SUMOylated at Lysine169 and at a second promiscuous site

**Figure 2:**
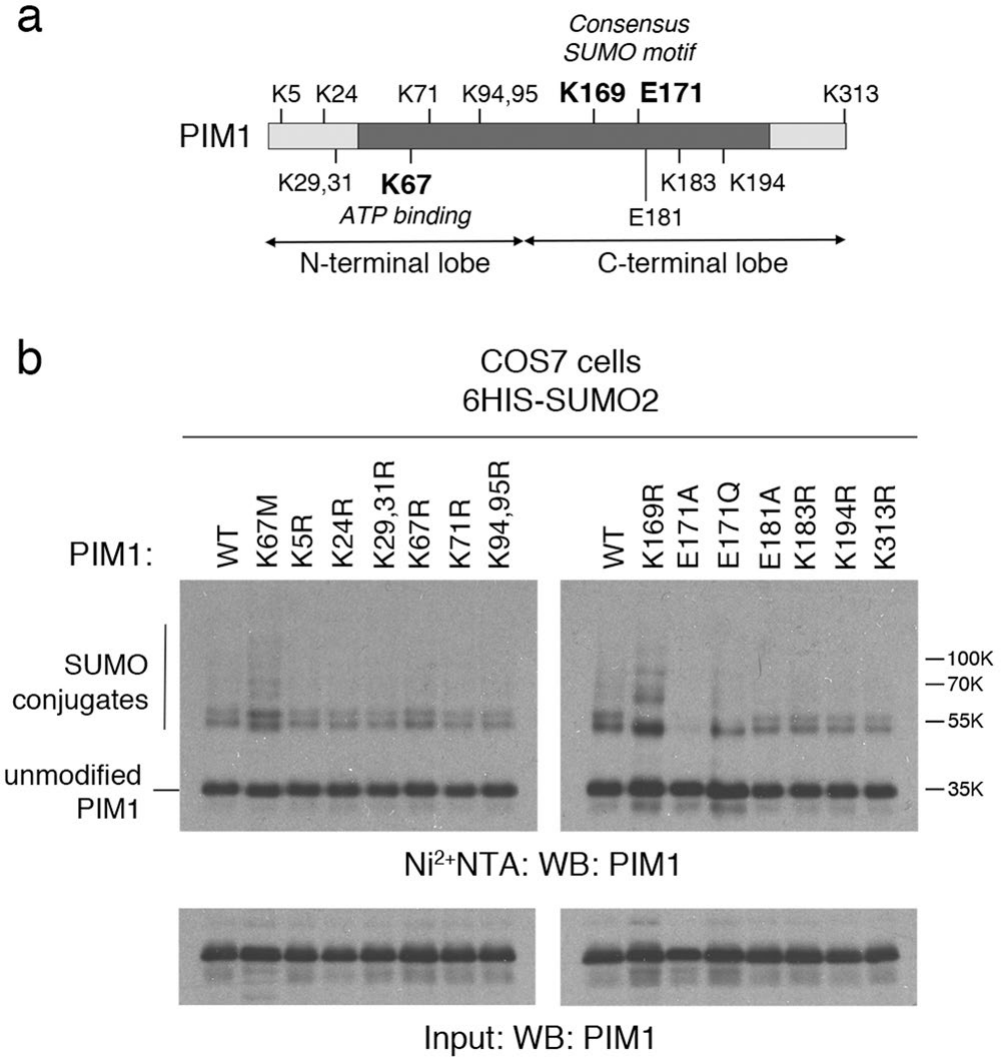
Identification of the sites of SUMOylation in PIM1. (**a**) Schematic showing the kinase domain of PIM1, and the position of various lysine (K) and and glutamic acid (E) residues predicted to be involved in SUMOylation. (**b**) WT PIM1 or single amino acid substitution site mutants were expressed at near equal protein levels in COS7 cells with 6His-SUMO2, by transfecting different amounts of plasmids. Empty vector was included, where appropriate, to maintain equal amounts of transfected plasmid DNA. An aliquot of whole cell lysate was taken as input, and the remainder subjected to Ni^2+^-NTA pull-down to capture SUMOylated proteins. The samples were subjected to SDS-PAGE followed by western blotting using a PIM1 antibody.

To determine whether K169 (within the consensus SUMO-motif: Fig. 2a) could act as a SUMO-acceptor, an arginine substitution mutant was generated using site-directed mutagenesis. SUMOylation assays were carried out using WT PIM1 and the K169R PIM1 mutant, co-expressed with 6His-SUMO2 (Fig. 2b). As observed previously, SUMOylated PIM1 (WT) ran as a doublet of approximate molecular weight 55 kDa. Interestingly, the K169R mutant was still capable of undergoing SUMOylation, but only a single species at 55 kDa molecular weight was observed. This suggests that K169 is a major site of SUMOylation, but that another non-consensus site can be SUMOylated independently of K169.

Consensus SUMOylation sites are also characterized by the presence of an invariant glutamic acid at the +2 position relative to the acceptor lysine residue. Strikingly, alanine substitution of the consensus glutamic acid residue within the postulated SUMOylation motif in PIM1 (E171A) abolished SUMOylation of the protein, suggesting that this residue is critical for overall SUMOylation of PIM1, irrespective of the target residue(s). Curiously, a conservative E171Q substitution only partially eliminated PIM1 SUMOylation similar to the K169R mutant. E181 was also mutated to alanine as it formed an inverted SUMO motif (D/ExKΨ) with lysine 183 (K183). However, SUMOylation of this mutant was similar to that of wild type (WT) PIM1. Arginine substitution of each of the remaining 11 lysines in PIM1 did not reveal any other specific site of SUMO modification, including the K67M mutant, which is catalytically inactive^17^, indicating that SUMOylation of PIM1 occurs independently of its protein kinase activity. Overall, these analyses identify K169 as a specific site of SUMO modification in PIM1, and suggest that there is a second (non-consensus) site(s) of modification, which appears to be promiscuous. The SUMOylation status of these mutants, together with their functional properties (see below) is shown in Table 1.

**Table 1:** SUMOylation status and functional characteristics of PIM1 mutants.

PARAMETER	WT Pim1	K169R	E171A	K67M
SUMOylation	++	+	−	++
Autophosphorylation	+	−	+	−
*In vitro* Histone H3 Kinase activity	++	++	++	−
*In vitro* c-MYC Kinase activity	++	+	+	−
Bad phosphorylation in U2OS	+	−	−	−
Histone H3 phosphorylation in U2OS	+	+	+	−
Deactivation of Akt in U2OS	+	+	+	−
Activation of ERK1/2 in U2OS	+	−	−	−
Stability	++	+	+++	+
Ubiquitylation	++	++	+	++
Localization	No change	No change	No change	No change

### PIAS1 and PIAS3 can act as E3 SUMO ligases for PIM1

Members of the Protein Inactivator of Activated STATs (PIAS) family, which negatively regulate the JAK-STAT pathway, were the first E3 SUMO ligases to be identified^18, 19^. Interestingly, PIM1 itself is a downstream target of the JAK-STAT pathway, which led us to hypothesize that PIAS proteins could function as E3 SUMO ligases for PIM1.

**Figure 3:**
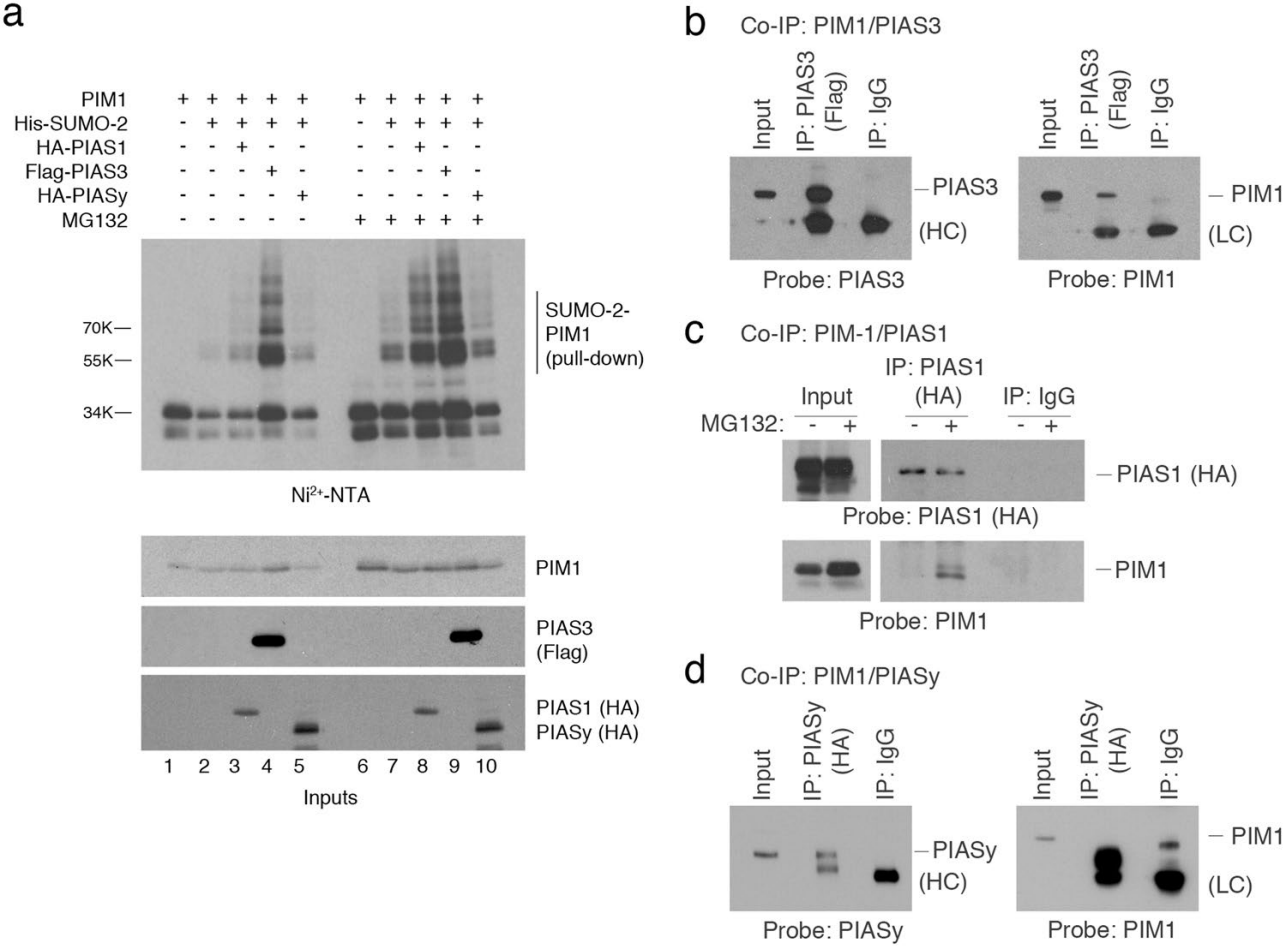
PIAS family members can directly interact with PIM1, and act as E3 SUMO ligases for PIM1. (**a**) Western blots showing SUMOylation assay performed in COS7 cells transfected with plasmids expressing PIM1, 6His-SUMO2 with PIAS1, PIAS3 or PIASy in the absence or presence of MG132 (20 μM for 6 hours). A western blot of whole cell lysate (input) was also performed to confirm the expression PIM1, PIAS1, PIAS3 and PIAS3 using the indicated antibodies. (**b**) H1299 cells were co-transfected with PIM1 and PIAS3 expression plasmids, and co-immunoprecipitation was performed using anti-Flag-antibody to pull-down PIAS3 associated complexes. The immunoprecipitated (IP) samples were analyzed by western blotting using Flag-tag and PIM1 (12H8) antibodies. Mouse IgG was used as a negative control. (**c**) H1299 cells were transfected with plasmids expressing PIM1 and PIAS1 in the presence or absence of MG132 (20 μM for 6 hours), and co-immunoprecipitation was performed using HA-tag (PIAS1) antibody. The IP samples were western blotted for the presence of PIAS1 and PIM1 using anti-HA- and PIM1 (12H8) antibodies. Mouse IgG was used as a negative control. (**d**) H1299 cells were co-transfected with PIM1 and PIASy expression plasmids, and co-immunoprecipitation was performed using anti-HA-antibody to pull-down PIASy associated complexes. The immunoprecipitated (IP) samples were analyzed by western blotting using anti-HA (PIASy) and PIM1 (12H8) antibodies. Mouse IgG was used as a negative control.

To test this idea, SUMOylation of PIM1 was examined in COS7 cells transfected with plasmids expressing WT PIM1, 6His-SUMO2, and either PIAS1, PIASy (also called PIAS4) or PIAS3. As above, SUMOylation of PIM1 (with SUMO2) by endogenous modifying enzymes could be observed (Fig. 3a, lanes 2 and 7). Under basal conditions, only PIAS3 greatly enhanced PIM1 SUMOylation suggesting that it can act as an E3 SUMO ligase for PIM1 (Fig. 3a, lanes 4 and 9). Treatment of the cells with the proteasome inhibitor, MG132, led to an increase in the levels of PIM1 SUMOylation even in the absence of an E3 SUMO ligase suggesting that SUMOylated PIM1 is targeted for degradation by the proteasome. Strikingly, PIAS1 could also stimulate PIM1 SUMOylation under these conditions (Fig. 3a, lanes 7 and 8), although to a lesser extent than PIAS3. In contrast, no such stimulation was seen when PIASy was used (Fig. 3a, lanes 7 and 10). Further analysis by co-immunoprecipitation demonstrated that PIM1 and PIAS3 associate, consistent with their interaction within the cell (Fig. 3b). Co-immunoprecipitation of PIM1 with PIAS1 was also observed but only when the cells had been treated with MG132 (Fig. 3c). No association could be detected between PIM1 and PIASy (Fig. 3d).

### SUMOylation increases the protein kinase activity of PIM1 *in vitro*

**Figure 4:**
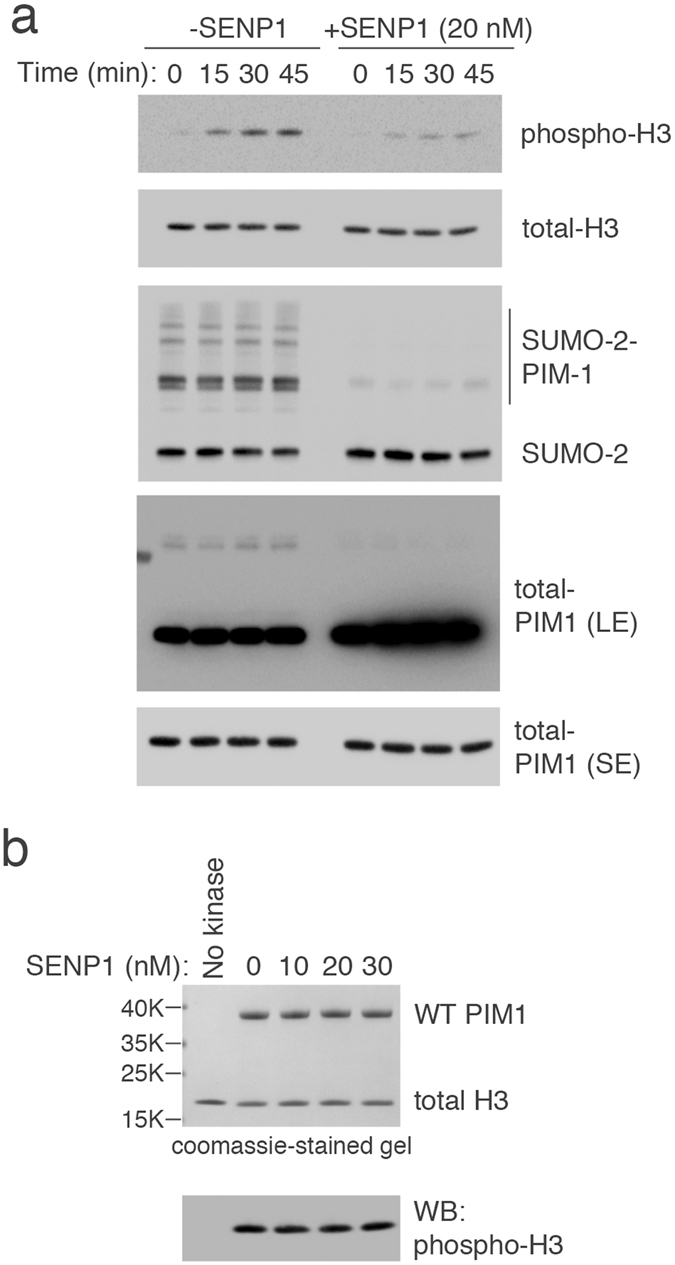
SUMOylation increase PIM1 kinase activity *in vitro*. (**a**) Bacterially purified 6His-PIM1 was SUMOylated *in vitro* using purified GST-SUMO2. Equal amounts of SUMOylated protein (including PIM1) were captured using GST-beads and incubated without or with SENP1 catalytic domain for 1 hour at 30 °C. Kinase assays were then performed using Histone H3.3 as a substrate for at 30 °C for 0, 15, 30 and 45 min. Kinase activity of SUMO2-modified or unmodified PIM1 was measured by analyzing Histone H3.3 phosphorylation using a phospho-specific antibody. Equal levels of substrate and kinase were confirmed by western blotting using indicated antibodies. (**b**) Purified WT PIM1 was first incubated with or without SENP1 catalytic domain fragment for 1 hour at 30 °C, and immediately used in a kinase assay using Histone H3.3 as substrate for 30 min at 30 °C. PIM1 kinase activity was measured by analyzing Histone H3.3 phosphorylation using a phospho-specific antibody. Equal levels of substrate and kinase were confirmed by coomassie staining of the gel.

The location of K169 in the active site of PIM1 suggests that a SUMO moiety could either inhibit kinase activity by sterically blocking access to the substrate, or promote kinase activity by regulating kinase-substrate interactions. To explore this idea, *in vitro* protein kinase assays were carried out using purified 6His-PIM1 that had been SUMOylated with GST-tagged SUMO2 *in vitro* and subsequently affinity-purified for analysis (Supplementary Fig. 1). For an accurate assessment of relative protein kinase activity, equal amounts of SUMOylated PIM1 were compared with PIM1 that had been de-SUMOylated *in vitro* using the catalytic domain (aa 415–643) of the SUMO protease SENP1^20^. The data (Fig. 4a) indicate that SUMOylated PIM1 was significantly more active in phosphorylating Histone H3.3 at Ser10 when compared with unmodified PIM1 at all time points. Western blotting for total Histone H3.3 confirmed equal amount of substrate in these reactions. Similar results were obtained when BAD was used as a substrate (Supplementary Fig. 2).

To rule out that SENP1 affected PIM1 protein kinase activity, unmodified PIM1 was first incubated with increasing concentrations of SENP1 catalytic domain followed by kinase assay. However, no obvious difference was noted in the level of phosphorylation of Histone H3.3 substrate in the absence or presence of the SENP1 fragment (Fig. 4b) confirming that SUMOylated PIM1 is more active towards Histone H3.3 than unmodified PIM1.

### Key residues required for SUMOylation selectively impact the activity and substrate specificity of PIM1

**Figure 5:**
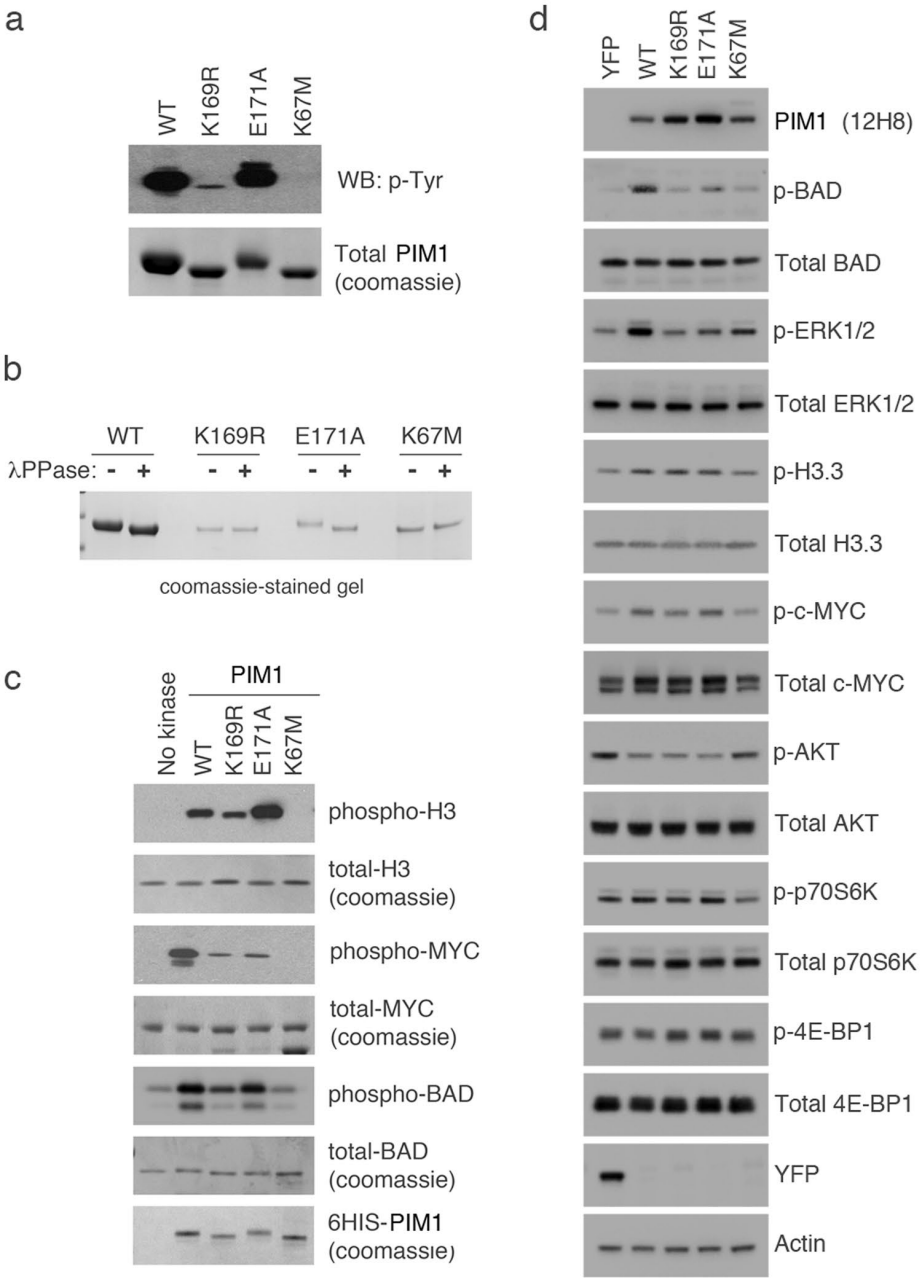
PIM1 SUMOylation regulates substrate specificity *in vitro* and in cultured cells. (**a**) 6His-PIM1 (WT or mutant) was expressed and purified from bacterial cells, and resolved by SDS-PAGE. A western blot for the same samples was also performed using a pan-phospho tyrosine antibody to detect PIM1 autophosphorylation. (**b**) The purified 6His-PIM1 proteins were treated with lambda phosphatase (+) to remove overall phosphorylation or untreated (−). Samples were resolved by SDS-PAGE, and stained with coomassie to visualize a shift in mobility, which is indicative of dephosphorylation. (**c**) *In vitro* kinase assays were carried out using recombinant c-MYC or Histone H3.3 as substrates, in the absence or presence of the indicated purified 6His-PIM1 proteins. The samples were resolved by SDS-PAGE, and either stained with coomassie to detect total protein levels or transferred to a nitrocellulose membrane for western blotting using phospho-specific antibodies as a measure of PIM1 kinase activity. (**d**) U2OS-FRT cells expressing YFP alone, YFP-WT PIM1 and YFP-E171A were treated with 10 ng/ml doxycycline; U2OS-FRT expressing YFP-K169R was treated with 20 ng/ml doxycycline and U2OS-FRT expressing YFP-K67M was treated with 50 ng/ml doxycycline for 48 hours, followed by western blotting using indicated antibodies.

Prior to investigating the intracellular effects of substituting key residues in PIM1 that are required for SUMOylation (K169R, E171A), mutant proteins were expressed in bacteria as 6His-PIM1 proteins, affinity-purified, and tested to determine whether they retained protein kinase activity. PIM1 can autophosphorylate on serine, threonine and tyrosine^21, 22^. SDS-PAGE of the purified proteins revealed a distinguishable mobility shift of WT PIM1 to an apparent higher molecular weight (characteristic of a phosphorylation event) as compared with the K67M inactive mutant, which cannot autophosphorylate^17^ (Fig. 5a, lower panel). The mobility of the K169R mutant was identical to the K67M mutant, suggesting impairment of its autophosphorylation function. In contrast, the E171A mutant, like WT PIM1, remained active. Treatment of the purified proteins with lambda phosphatase confirmed that the difference in mobility was due to autophosphorylation of PIM1 (Fig. 5b). Western analysis using a pan-phosphotyrosine antibody (Fig. 5a, upper panel) also indicated that only WT PIM1 and the E171A mutant could autophosphorylate.

To determine whether the K169R and E171A mutants were catalytically active on established PIM1 substrates, *in vitro* protein kinase assays were performed. Western blotting analysis confirmed that that WT, K169R and E171A PIM1 proteins, but not the K67M mutant, were active against Histone H3.3 (pSer10^23^) (Fig. 5c), although less active in the case of the K169R mutant. Similar results were seen when BAD (pSer112^24^) was used as substrate. Both E171A and K169R could phosphorylate c-MYC (pSer62^25^) but at a lower level as compared with WT PIM1. These data suggested that the E171A mutant largely retained activity but that generally the K169R mutant was catalytically impaired.

To explore loss of SUMOylation under cellular conditions, the phosphorylation status of PIM1 substrates was examined in U2OS-FRT cells inducibly expressing YFP alone or YFP-PIM1 (WT or mutant) (Supplementary Fig. 3a,b). The data (Fig. 5d) suggest that WT PIM1, but not the K67M mutant, is active towards Histone H3 at Ser10, c-MYC at Ser62, BAD at Ser112 and ERK1/2 at Thr202/Tyr204 (Note that activation of ERK1/2 is an indirect effect of PIM1 activation in cells^26^). No apparent activity was noted towards p70S6K or 4E-BP1. The K169R mutant showed little or no activity towards BAD, ERK1/2 and c-MYC, thereby reflecting its *in vitro* activity towards two of these substrates (BAD and c-MYC), and its autophosphorylation, suggesting that K169 may indeed entail an important catalytic function. The E171A mutant showed impaired activity towards BAD and ERK1/2, consistent with a role in activation by SUMOylation, but did not show any reduced activity towards c-MYC, possibly reflecting other cellular events affecting its substrate selectivity. Both mutants, like WT PIM1, showed a slight stimulation of Histone H3.3 Ser10 phosphorylation. Curiously, WT PIM1 and the two SUMO mutants reduced the levels of phospho (active)-AKT reflecting a compensatory adjustment since these two protein kinase share many substrates^27^. We have not been able to observe any obvious phenotypes resulting from PIM1 expression in these cells (data not shown). Previous studies have suggested that overexpression of PIM1 may be superfluous in cells already expressing all PIM isoforms^9^. Taken together, these findings suggest that SUMOylation may regulate the phosphorylation of selected substrates by PIM1 although, as stated above, it is difficult to rule out possible catalytic consequences of the mutations.

### PIM1 that cannot be SUMOylated is significantly more stable than WT PIM1

**Figure 6:**
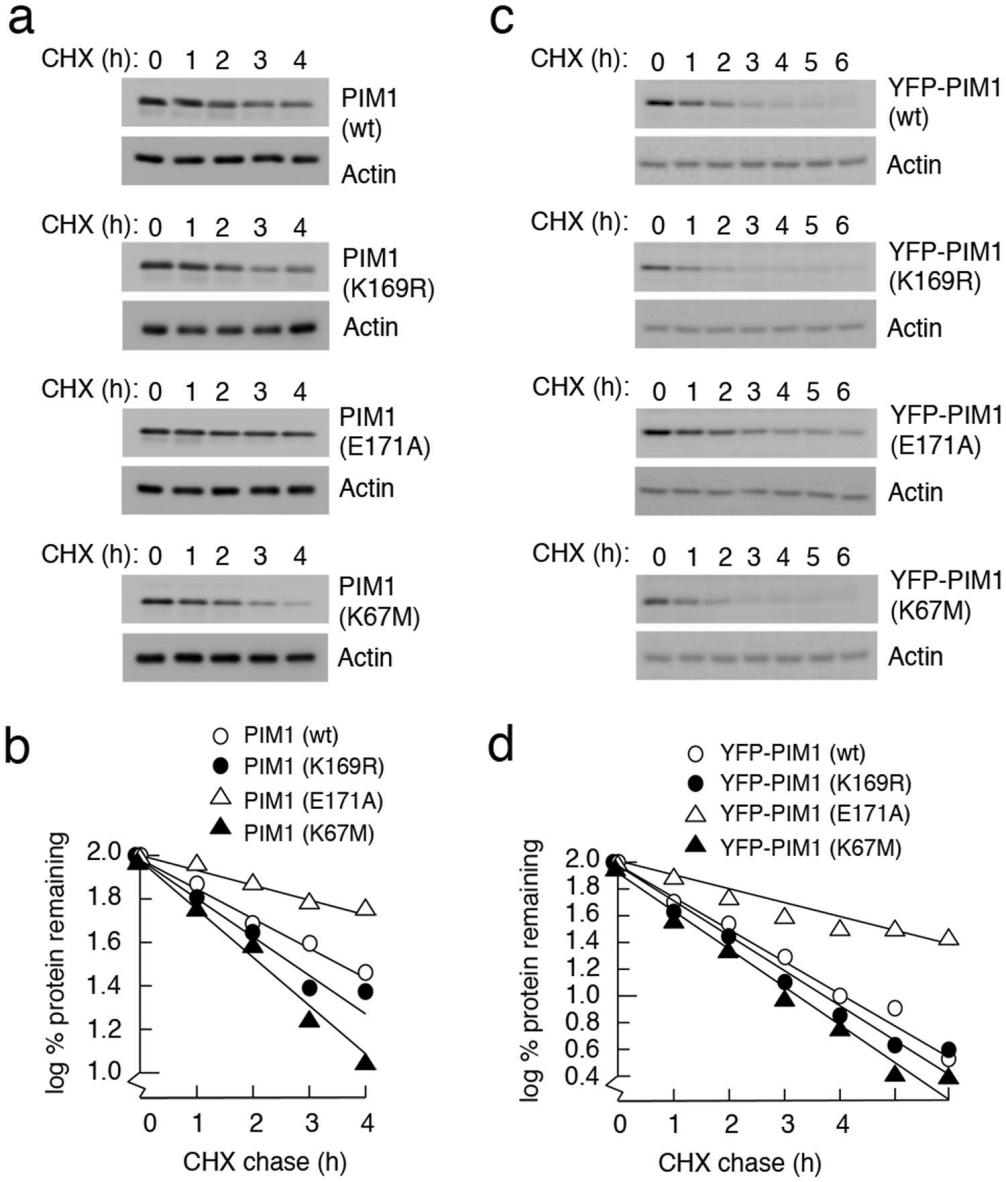
SUMOylation regulates PIM1 stability. (**a**) Plasmids encoding WT MYC-PIM1, PIM1 SUMO mutants (MYC-K169R and MYC-E171A) and a catalytically inactive mutant (MYC-K67M) were transiently transfected into H1299 cells. Cycloheximide was added to the cells 24 hours post transfection at a final concentration of 50 μg/ml to inhibit protein synthesis, and harvested at the indicated time points. The cell lysates were analyzed by western blotting using PIM1 (12H8) antibody. Actin was used as a loading control. (**b**) PIM1 band intensity in each case was quantified relative to the zero time point, using Biorad ImageLab software, and plotted on a graph as percentage of protein remaining in log scale. (**c**) HeLa-FRT cells expressing YFP-tagged WT PIM1 or mutant PIM1 were treated with 50 ng/ml doxycycline for 24 hours to induce protein expression, following which cycloheximide chase assay was performed as done in panel A. (**d**) PIM1 protein levels were quantified and represented graphically as done in panel B.

Next, we examined the potential impact of SUMOylation on PIM1 protein stability by cycloheximide-chase analysis in H1299 cells transiently transfected with PIM1 (WT or mutant) plasmids. The half lives of the WT, K169R and K67M PIM1 proteins were approximately 120, 90, and 75 min respectively (Fig. 6a and b). However, the E171A mutant appeared to be much more stable with a half-life of greater than 4 h.

To rule out that these effects were due to differences in transfection efficiencies or expression levels, cycloheximide-chase analysis was carried out using single-copy isogenic HeLa-FRT-derived lines that permitted inducible expression of WT and mutant PIM1 proteins as YFP-fusion proteins (Supplementary Fig. 3c,d). In this system the differences in turnover were qualitatively similar to those observed following transient expression, with the E171A mutant showing a significant increase in stability (Fig. 6c and d). These data suggest that SUMOylation is required for efficient turnover of PIM1, although we cannot rule out the possibility that E171 may play a more direct role in engaging the protein degradation machinery. The rapid turnover of the K169R mutant, which can still be partially SUMOylated, may be associated with loss of auto-catalytic activity, which has been previously linked to reduced PIM1 stability^10, 11^.

### SUMOylation downregulates PIM1 levels by promoting RNF4-dependent ubiquitylation and proteasomal degradation

**Figure 7:**
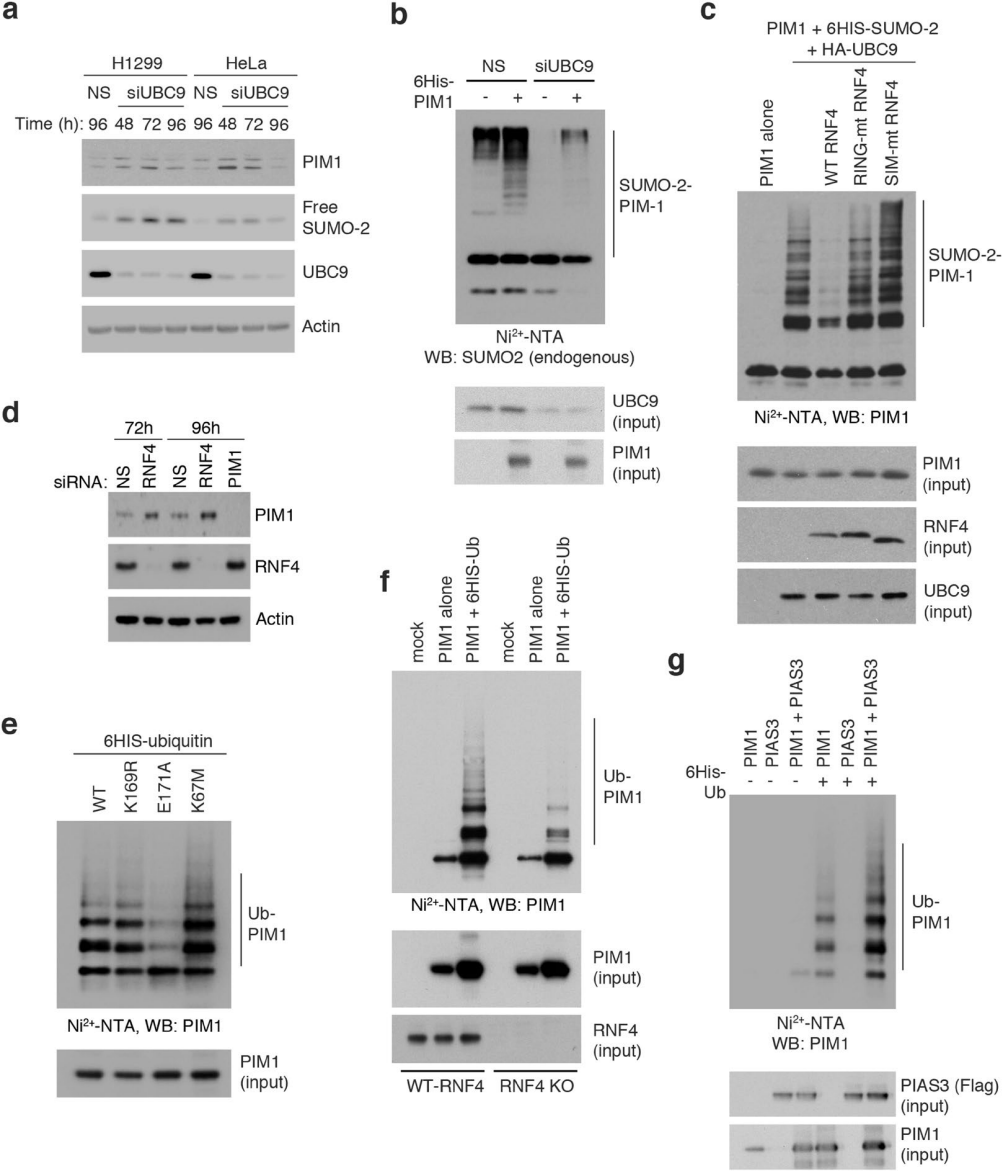
SUMOylation negatively regulates PIM1 protein levels by promoting its ubiquitylation and proteasomal degradation via RNF4. (**a**) H1299 or HeLa cells were transfected with siRNA targeting UBC9 (siUBC9), or non-targeting siRNA (NS) as negative control. Lysates were harvested at the indicated time points, and western blotting was performed for endogenous PIM1 (using 12H8 antibody), SUMO2 and UBC9. Actin was used as a loading control. (**b**) H1299 cells were transfected with a plasmid expressing 6His-PIM1 in the absence and presence of Ubc9 siRNA, and PIM1 was affinity purified under denaturing conditions as done previously for 6His-SUMO protein. Eluted proteins were analyzed by western blotting using SUMO2 antibody to detect SUMOylated PIM1. (**c**) SUMOylation assay was done in COS7 cells transfected with plasmids expressing MYC-tagged PIM1 alone or with HA-UBC9 and 6His-SUMO2 to stimulate PIM1 SUMOylation. Cells were additionally transfected with plasmids expressing WT RNF4 or RING finger mutant RNF4 (mRING) or SUMO-interaction motif mutant RNF4 (mSIM). PIM1 SUMOylation was analyzed by western blotting of Ni^2+^-NTA pull-down samples using PIM1 (12H8) antibody. Western blotting was also performed on the input samples using indicated antibodies to check expression of transfected proteins. (**d**) H1299 cells were transfected with siRNA targeting RNF4, PIM1, or non-targeting siRNA (NS) as negative control for the indicated time points. Western blotting was performed for endogenous PIM1 (using 12H8 antibody) and RNF4. Actin was used as a loading control. (**e**) Ubiquitylation assay was performed in H1299 cells transfected with plasmids expressing MYC-tagged WT PIM1, K169R, E171A and K67M with 6His-ubiquitin. Ubiquitylated PIM1 was detected by western blotting using 12H8 (PIM1) antibody. (**f**) Ubiquitylation assay was performed in parental H1299 cells, or H1299 RNF4 knock-out cells, transfected with plasmids expressing MYC-tagged WT PIM1 and 6His-ubiquitin. Ubiquitylated PIM1 was detected by western blotting using PIM1 (12H8) antibody. (**g**) Ubiquitylation assay was performed in H1299 cells transected with plasmids expressing MYC-tagged WT PIM1 and Flag-tagged PIAS3, with or without 6His-ubiquitin. Ubiquitylated PIM1 was detected by western blotting using PIM1 (12H8) antibody.

To further investigate the role of SUMOylation in regulating PIM1 turnover, we silenced UBC9 expression (the sole SUMO E2 enzyme) in H1299 and HeLa cells. In both cases, decreased levels of UBC9 were accompanied by increased levels of endogenous PIM1 (Fig. 7a), thereby supporting a role for SUMO modification in promoting PIM1 turnover. However, we cannot rule out that this is an indirect effect as changes in UBC9 levels also affect global SUMOylation in cells. When 6His-tagged PIM1 was expressed in H1299 cells and subsequently isolated on Ni^2+^-agarose beads, a series of high molecular weight bands was observed, consistent with multiple attachments of SUMO and/or ubiquitin. These bands disappeared following silencing of UBC9 expression (Fig. 7b), consistent with the idea that UBC9 promotes the appearance of poly-SUMO- or SUMO-ubiquitin hybrid chains on PIM1.

SUMOylation can promote ubiquitin-mediated degradation of some substrates by recruiting the SUMO-targeted E3 ubiquitin ligase, RNF4^28^. To test this idea, PIM1 was transiently expressed in COS7 cells, together with 6His-SUMO2 and UBC9 to induce polySUMOylation, and in the presence or absence of WT or mutant RNF4 proteins. SUMOylation assay (Fig. 7c) confirmed that expression of SUMO2 and UBC9 with PIM1 led to the formation of polySUMOylated PIM1. Co-expression of RNF4 caused a decrease in the levels of polySUMOylated PIM1. This effect was most likely due to selective ubiquitin-mediated degradation of polySUMOylated PIM1, as mutant forms of RNF4, where either the RING domain (mRING) or the SUMO interaction motifs mSIM) were mutated did not lead to disappearance of SUMOylated PIM1. Additionally, silencing of endogenous RNF4 caused a substantial increase in endogenous PIM1 levels (Fig. 7d). These observations support that idea that SUMOylation of PIM1 acts as a signal for RNF4-mediated ubiquitylation, leading to proteasomal degradation.

We also performed ubiquitylation assays in H1299 cells co-transfected with PIM1 and 6His-tagged ubiquitin plasmids. Western blot analysis of the captured proteins showed that the WT, K169R and K67M PIM1 proteins were ubiquitylated to similar extents (Fig. 7e). However, the ubiquitylation of the E171A mutant was significantly reduced relative to WT PIM1. Since E171A can only be SUMOylated very weakly (Fig. 2b) these data support that idea that SUMOylation can promote PIM1 ubiquitylation. To confirm that RNF4 is an E3 ubiquitin ligase for PIM1, we performed ubiquitylation assays in parental H1299 cells and in H1299 cells in which RNF4 had been knocked out by CRISPR. The data indicate that the ubiquitylation of PIM1 was significantly reduced in the RNF4 knock-out cells as compared with the parental line (Fig. 7f). Ubiquitylation of PIM1 was also examined in the absence and presence of co-expressed PIAS3. As shown in Fig. 7g, PIAS3 also stimulated the ubiquitylation of PIM1. Taken together, these experiments support a model in which SUMOylation of PIM1 can lead to the subsequent ubiquitylation and degradation of PIM1.

## Discussion

In the present study we have established that SUMOylation regulates two independent functions of PIM1. Firstly, we have shown that SUMOylation mediates RNF4-dependent ubiquitylation and proteasomal turnover of PIM1. We cannot rule out at this stage, however, that other mechanisms may also operate to mediate PIM1 turnover. Secondly, our data indicate that SUMOylation of purified PIM1 *in vitro* directly stimulates its protein kinase activity towards two established PIM1 substrates: Histone H3 (Ser10) and BAD (Ser112). It is challenging to confirm unequivocally that this regulation occurs within a cellular context as it is not possible to mimic PIM1 SUMOylation in cultured cells. Moreover, under steady state conditions only a small proportion of molecules are SUMOylated at any given time^14, 29^. Nevertheless, an alanine substitution mutant of E171 (a key amino acid in PIM1 required for SUMOylation to occur) is fully active *in vitro* yet shows a significant reduction in BAD-Ser112 phosphorylation in cells. This is consistent with loss of the ability of this mutant to undergo SUMOylation. These two novel observations establish for the first time, to our knowledge, that PIM1 can be regulated through non-autocatalytic post-translational modification events. Further experimentation did not reveal any detectable changes arising from loss of SUMOylation in subcellular localization (Supplementary Fig. 4), association with partner/substrates proteins such as c-MYC (Supplementary Fig. 5), or the ability to stimulate c-MYC-dependent transcription (Supplementary Fig. 6). However, it is yet possible that there are other PIM1-associated functions that may be affected by this modification.

**Figure 8:**
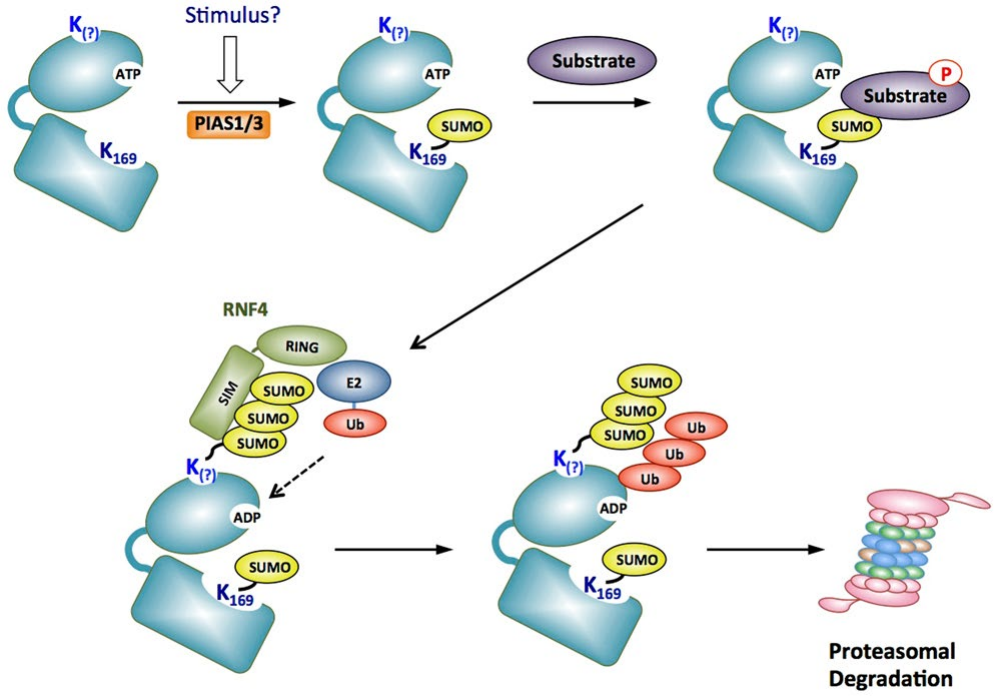
Model for regulation of PIM1 by SUMOylation. The bi-lobed structure of PIM1 kinase is shown in blue. The consensus SUMOylated lysine residue is located in the substrate binding pocket (K169 in deep blue). For the purpose of presentation, the non-consensus lysine is shown in blue in the N-terminal domain. Stimuli such as growth factors or stress might induce SUMOylation of PIM1 under endogenous conditions. SUMOylated PIM1 can bind and phosphorylate substrates. Once this is achieved, a SUMO targeted ubiquitin ligase is recruited to polySUMOylated PIM1 leading to attachment of polyubiquitin chains on PIM1. The SUMOylated and ubiquitylated PIM1 is then targeted for degradation by the proteasome.

The idea that SUMOylation increases the protein kinase activity of PIM1, yet also increases its degradation, may appear initially to be counter-intuitive. One plausible model to explain this, however, would be that, similar to the “ubiquitin clock” model proposed for certain transcription factors such as MYC^30^, the activity of PIM1 may be governed by the length of time it takes to transition from the SUMOylated and active form to a SUMOylated and ubiquitylated form that is destroyed by the proteasome. A model for the regulation of PIM1 by SUMOylation, consistent with this idea, is shown in Fig. 8. Additionally, it is known that other activated protein kinases can be degraded by the ubiquitin proteasome system or through the lysosomal pathway in cells. For example, a study performed with Protein kinase C suggested that the active conformation is necessary to elicit its degradation^31, 32^. Moreover, activation of the receptor tyrosine kinase, epidermal growth factor receptor (EGFR) by its ligand, epidermal growth factor (EGF) was shown to not only activate its kinase activity, but also trigger its degradation^33, 34^. Therefore, although PIM1 is constitutively active, the idea that SUMOylation can further enhance its activity, yet also result in its degradation, is consistent with these examples.

We were able to detect SUMOylation of endogenous PIM1 in cultured cells upon proteasome inhibition (Fig. 1) but have been unable to observe any changes in this modification in response to agents/conditions known to affect SUMOylation including DNA damage (induced by etoposide treatment) or proteotoxic stress (induced by thapsigargin or tunicamycin). Changes in the SUMOylation status of PIM1 may occur only within a selected or localized pool of PIM1, or in response to a particular cytokine or growth factor. Moreover, SUMOylation of PIM1 may lead to rapid protein degradation making it difficult to distinguish between SUMOylated and ubiquitylated PIM1 by western blotting of the total cellular PIM1 pool. Alternatively, SUMOylation-mediated turnover may serve as a normal house-keeping function. Nevertheless, alteration or impairment of the mechanisms we have identified may be a factor in human cancer development and could therefore hold opportunities for enhancing drug targeting of PIM1 kinase activity in cancer treatment.

Our data add to a growing body of evidence that SUMOylation of kinases may regulate at least a subset of these important enzymes. For example, SUMOylation of pancreatic Glucokinase^35^, AKT^36–38^, AMPKβ2^39^ and Protein kinase R^40^ stimulates their abilities to phosphorylate their respective substrates. Conversely, SUMOylation inhibits other kinases such as MEK^41^ and AMPKα1^42^. These different outcomes suggest that there is no unifying mechanism by which SUMOylation regulates kinase activity. Additionally, studying the effect of SUMOylation is particularly challenging in cases such as PIM1 when the modified lysine is present in the kinase domain, because it becomes difficult to distinguish between the functions of the lysine residue associated with the intrinsic activity of the kinase and SUMOylation of the substrate. In this respect, our data from the analysis of mutating E171, which is not an essential catalytic component but is critical for SUMOylation, provide a more reliable indication of the likely outcome of blocking SUMOylation. In spite of these challenges, future investigation of the role played by this regulatory mechanism in influencing the oncogenic function of PIM1 may provide a deeper understanding of the relationship of PIM1 to cancer development together with potential ideas for exploitation.

## Materials and Methods

### Cell culture and reagents

COS7, H1299, DU145, HeLa-FRT and U2OS-FRT were grown in DMEM medium and K562 cells were cultured in RPMI-1640 medium, both supplemented with 10% FBS (Biosera) and 2 mM L-glutamine (Thermo Fisher Scientific). Parental H1299 cells and H1299 cells in which RNF4 had been knocked out using CRISPR were grown in RPMI-1640 medium supplemented with 10% FBS (Biosera) and 2 mM L-glutamine (Thermo Fisher Scientific). These cells were a kind gift from Prof Ron Hay (University of Dundee). A description of the method used to generate this line is described elsewhere^43^.

### Antibodies and Western blotting

SDS-PAGE and western blotting was carried out using standard methods. The following antibodies were used: PIM1 (12H8, Santa Cruz), PIM1 (A300–313A, Bethyl Laboratories), Actin (ab6276, Abcam), GAPDH (G8795, Sigma), SUMO2 (51–9100, Zymed), His-tag (27-4710-01, GE Healthcare), HA-tag (12CA5, Sigma), Flag-tag (F1804, Sigma), MYC-tag (9E10, Hybridoma supernatant), GFP-tag (sc-8334, Santa Cruz), GST-tag (sc-459, Santa Cruz), total ERK1/2 (ER16, Transduction lab), and phospho S10 Histone H3 (06–570, Millipore). The following antibodies were purchased from Cell Signaling Technology: phospho S62 c-MYC (13748), total c-MYC (5605), total Histone H3 (4499), phospho S112 Bad (5284), total Bad (9239), phospho T37/46 4E-BP1 (2855), total 4E-BP1 (9644), phospho T389 p70S6K (108D2), total p70S6K (49D7), phospho S473 AKT (D9E), total AKT (40D4), phospho T202/Y204 ERK1/2 (9106) and phospho tyrosine-100 (9411). Sheep polyclonal UBC9 and chicken polyclonal RNF4 were from Ron Hay, University of Dundee. Secondary antibodies were purchased from Biorad and Thermo Fisher Scientific.

### Plasmids and transfection

Plasmids expressing 6His-SUMO1, 6His-SUMO2, 6His-SUMO3, HA-PIAS1, HA-PIASy, Flag-PIAS3 have been described previously^44^. Plasmids expressing WT MYC-PIM1, 6His-ubiquitin and GST-PIM1 were generated previously^45^. Rat WT RNF4, RNF4 SIM mutant (mSIM) and RNF4 RING mutant (mRING) and human UBC9 in pcDNA3 were from Ron Hay, University of Dundee. Plasmids expressing Flag-tagged WT SENP1 and mutant SENP1 (C602S) were provided by Laureano de la Vega, University of Dundee. Flag-tagged c-MYC plasmid was from Vicky Cowling, University of Dundee. The Myc-responsive 4X E-box promoter/pGL3 vector was a kind gift from Prof. Dr. Thorsten Berg, University of Leipzig.

To express YFP-tagged PIM1, cDNA encoding human PIM1 was amplified from MGC clone pOTB7-PIM1, and ligated into the EcoRI site of pEYFP-C1 vector (Clontech). YFP-PIM1 or YFP alone was PCR amplified from pEYFP-C1/PIM1 plasmid and ligated into the KpnI site of pcDNA5/FRT/TO vector (Thermo Fisher Scientific) for generation of tet-inducible cell lines. A 6His-PIM1 mammalian expression plasmid was generated by cloning PCR amplified PIM1 cDNA into the EcoRI site of pcDNA3 vector. The vector, pHAT2, used for the expression of 6His-PIM1 in bacterial cells was provided by Dr. Marko Hyvonen (University of Cambridge). The PIM1 cDNA was cloned into the EcoRI site of pHAT2. For retrovirus production, the PIM1 cDNA was cloned into the EcoRI site of pBABE-puro vector.

Plasmids were transfected into cells using Lipofectamine 2000 (Thermo Fisher Scientific) using DNA (μg) to Lipofectamine 2000 (μl) ratio of 1:3 according to manufacturer’s instructions.

### Site-directed mutagenesis

PIM1 mutants were generated using the QuikChange Lightning Site-directed Mutagenesis kit (Agilent Technologies) as per manufacturer’s instructions. The primer sequences have been described in Table 2. The coding region of all plasmids was sequenced to confirm the presence of the desired mutation.

**Table 2:** Primer sequences used for site-directed mutagenesis.

Primer name	Sequence (5′-3′)
PIM1 K5R For	TTC AAT GCT CTT GTC CAG AAT CAA CTC GCT TTG CCC
PIM1 K5R Rev	GGG CAA GCG AGT TGA TTC TGG ACA AGA GCA TTG AA
PIM1 K24R For	GGG CGC CAG CCT GGT GGC GTG CA
PIM1 K24R Rev	TGC ACG CCA CCA GGC TGG CGC CC
PIM1 K29, 31R For	AGG GGC TCC CTC TCC CTG CCG GGC GCC
PIM1 K29, 31R Rev	GGC GCC CGG CAG GGA GAG GGA GCC CCT
PIM1 K67R For	CTC CAC GTG TCT GAT GGC CAC CGG CAA GTT
PIM1 K67R Rev	AAC TTG CCG GTG GCC ATC AGA CAC GTG GAG
PIM1 K67M For	GTC CTT CTC CAC GTG CAT GAT GGC CAC CGG CA
PIM1 K67M Rev	TGC CGG TGG CCA TCA TGC ACG TGG AGA AGG AC
PIM1 K71R For	AAA TCC GGT CCC TCT CCA CGT GTT TGA TGG CCA
PIM1 K71R Rev	TGG CCA TCA AAC ACG TGG AGA GGG ACC GGA TTT
PIM1 K94, 95 R For	CCG AGC TCA CCC TCC TCA GCA GGA CCA CTT CCA TGG
PIM1 K94, 95 R Rev	CCA TGG AAG TGG TCC TGC TGA GGA GGG TGA GCT CGG
PIM1 K169R For	GTG CTC CAC CGC GAC ATC AGG GAC GAA AAC ATC
PIM1 K169R Rev	GAT GTT TTC GTC CCT GAT GTC GCG GTG GAG CAC
PIM1 E171A For	CCG CGA CAT CAA GGA CGC AAA CAT CCT TAT CGA CC
PIM1 E171A Rev	GGT CGA TAA GGA TGT TTG CGT CCT TGA TGT CGC GG
PIM1 E171Q For	GAG GTC GAT AAG GAT GTT CTG GTC CTT GAT GTC GCG GTG
PIM1 E171Q Rev	CAC CGC GAC ATC AAG GAC CAG AAC ATC CTT ATC GAC CTC
PIM1 E181A For	CGA TGA GCT TGA GCG CGC CGC GAT TGA GG
PIM1 E181A Rev	CCT CAA TCG CGG CGC GCT CAA GCT CAT CG
PIM1 K183R For	GAA GTC GAT GAG CCT GAG CTC GCC GCG ATT G
PIM1 K183R Rev	CAA TCG CGG CGA GCT CAG GCT CAT CGA CTT C
PIM1 K194R For	GAC GGT GTC CCT GAG CAG CGC CCC C
PIM1 K194R Rev	GGG GGC GCT GCT CAG GGA CAC CGT C
PIM1 K313R For	GAA AGG CTG CTA TCT GCT GGG CCC CGG
PIM1 K313R Rev	CCG GGG CCC AGC AGA TAG CAG CCT TTC

### siRNA transfection

These were carried out using Lipofectamine RNAiMAX reagent (Thermo Fisher Scientific) according to manufacturer’s instructions. A pool of 4 siRNAs (ON-TARGETplus SMARTpool, Dharmacon) was used to silence the expression of endogenous UBC9, RNF4 and PIM1 with Non-targeting siRNA pool as the negative control.

### *In vivo* SUMOylation and Ubiquitylation assay

SUMOylated or ubiquitylated proteins were pulled down from the cell lysate using Ni^2+^-NTA agarose beads (Qiagen) as described in the published protocol^28^.

### Co-Immunoprecipitation

These were carried out as described previously^46^.

### Recombinant protein expression and purification

6His-tagged or GST-tagged PIM1 proteins were expressed in Rosetta 2 (DE3) pLys cells (Novagen). Protein induction was achieved by IPTG addition (100 μM final concentration) and further incubation for 3–4 hours at 30 °C. The cell pellet was lysed in B-PER bacterial protein extraction reagent (Thermo Fisher Scientific) and affinity purified using Ni^2+^-NTA agarose beads (Qiagen) or GST-sepharose (GE Healthcare) using standard protocol. Proteins were stored in buffer containing 50 mM Tris pH 7.5, 500 mM NaCl and 1 mM DTT after elution and dialysis. Purified recombinant SUMO1 and 2, SAE1/2, UBC9, IR1 + M fragment of RanBP2 and SENP1 catalytic domain were a gift from Ron Hay, University of Dundee.

### *In vitro* protein kinase assay

PIM1 kinase assays were carried out using recombinant 6His-PIM1 (WT or mutant) with Histone H3.3 (M2507, NEB UK), c-MYC (MRC-PPU Reagents, University of Dundee) or BAD (SRP5164, Sigma) as substrate. Reactions were carried out in a total volume of 20 μl containing 50 mM Tris-HCl pH 7.5, 10 mM MgCl_2_, 0.2 mM ATP, 1 μg substrate and approximately 0.5–1 μg PIM1 kinase. Reactions were incubated at 30 °C for 30 min, and terminated by addition of 2X SDS sample buffer (with DTT). Phosphorylated proteins were detected by western blotting using phospho-specific antibodies.

### Small scale *in vitro* SUMOylation reactions

Experiments shown in Fig. 1a and b were carried out using procedure described previously^44^.

### Purification of SUMOylated PIM1 kinase from *in vitro* reaction

*In vitro* SUMOylation of 6His-PIM1 was carried out overnight at 37 °C in 100 μl reaction containing 50 mM Tris pH 7.5, 3 mM ATP, 5 mM MgCl_2_, 5 mM DTT, 20 μg 6His-PIM1, 50 μg GST-SUMO2, 5 μg UBC9, 0.9 μg SAE1/2 and 1.5 μg IR1 + M fragment of RanBP2. The reaction was diluted 5-fold with 50 mM Tris pH 7.5 and SUMOylated proteins were captured by adding 400 μl (50% slurry) glutathione sepharose beads (GE Healthcare) overnight at 4 °C. The beads were washed three times in wash buffer (50 mM Tris pH 7.5 and 150 mM NaCl) and eluted by adding 400 μl elution buffer (20 mM reduced glutathione, 50 mM Tris pH 8, 150 mM NaCl and 1 mM DTT) for 2 hours at 4 °C.

### DeSUMOylation coupled kinase assay

Elutions from *in vitro* SUMOylation reactions were divided into two equal parts: one with 20 nM active SENP1 catalytic domain and the other without SENP1. First, the reactions were incubated at 30 °C for 1 hour for deSUMOylation to occur, following which 5 μg Histone H3.3 (substrate) and 1x kinase buffer (50 mM Tris pH 7.5, 10 mM MgCl2, 0.2 mM ATP) was added. The reactions were further incubated at 30 °C for the kinase assay reaction, and aliquots were collected at 0, 15, 30 and 45 min for analysis of histone H3.3 phosphorylation by western blotting using a phospho-specific antibody.

### Lambda phosphatase treatment

Dephosphorylation of PIM1 was carried out using lambda protein phosphatase kit (NEB, UK) according to manufacturer’s instructions.

### Generation of tetracycline (tet) inducible cell lines

HeLa-FRT cells were a kind gift from Prof. Stephen Taylor (University of Manchester) and U2OS-FRT were a kind gift from Prof. Kevin Hiom (University of Dundee). Tet-inducible cell lines were generated according to Flp-In T-REx instruction manual (Thermo Fisher Scientific).

## Electronic supplementary material

Supplementary Information
