# Supplementary material for: A functional SUMO-motif in the active site of PIM1 promotes its degradation via RNF4, and stimulates protein kinase activity
Source: Sci Rep. 2017 Jun 15;7:3598. doi: 10.1038/s41598-017-03775-w (PMC5472562; doi:10.1038/s41598-017-03775-w)
Supplement: Supplementary file 1 — Supplementary Information [file 41598_2017_3775_MOESM1_ESM.pdf]

**SUPPLEMENTARY INFORMATION**

# A functional SUMO-motif in the active site of PIM1 promotes its degradation via RNF4, and stimulates protein kinase activity

R. Sumanth Iyer, Lynsey Chatham, Roger Sleigh and David W Meek

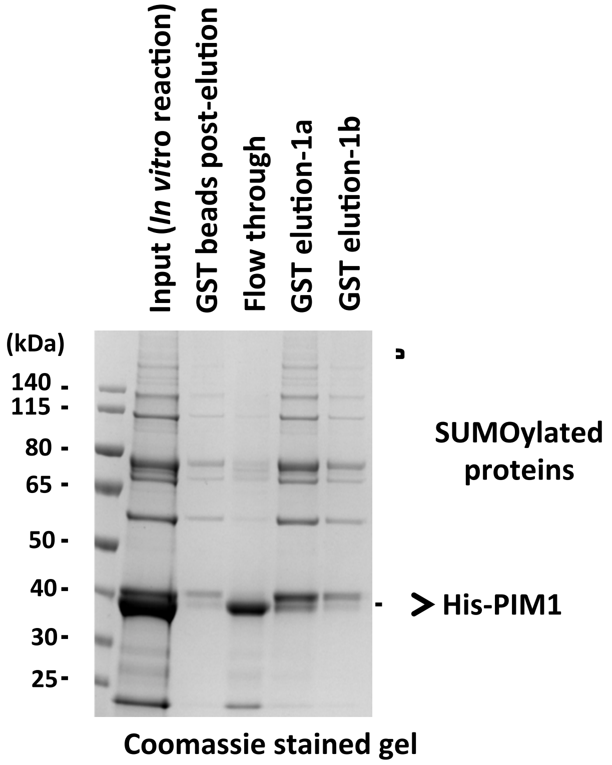


**Supplementary Figure 1.**

**Purification of GST-SUMOylated 6His-PIM1 from in vitro SUMOylation reaction.**

Products of the *in vitro* SUMOylation reactions were bound to glutathione-agarose-beads to precipitate all SUMOylated proteins including PIM1. The proteins were eluted from the beads using reduced glutathione. The samples were resolved by SDS-PAGE and stained with coomassie. (Subsequent affinity purification on Ni2+-NTA agarose to remove any other SUMOylated products, or free SUMO2, was explored but recovery from the beads was problematic and was therefore omitted.)

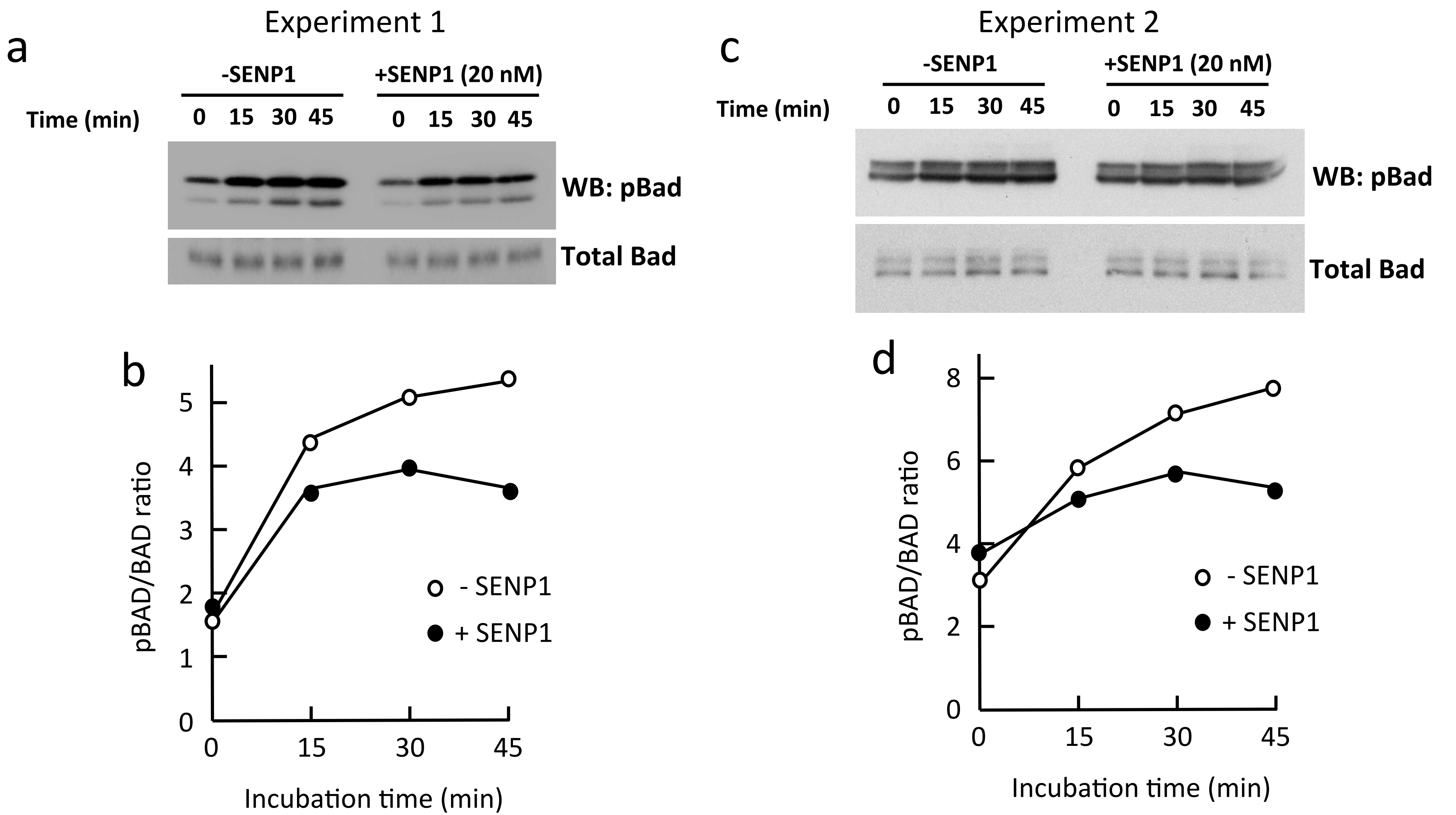


**Supplementary Figure 2. SUMOylation increase PIM1 kinase activity *in vitro.*** Bacterially purified 6His-PIM1 was SUMOylated *in vitro* using purified GST-SUMO2. Equal amounts of SUMOylated protein (including PIM1) were captured using GST-beads and incubated without or with SENP1 catalytic domain for 1 hour at 30 $^0$C. Kinase assays were then performed using BAD as a substrate for at 30 $^0$C for 0, 15, 30 and 45 min. Kinase activity of SUMO2-modified or unmodified PIM1 was measured by analyzing BAD phosphorylation using a phospho-specific antibody. Equal levels of substrate and kinase were confirmed by western blotting using indicated antibodies. **a,b** First of two separate experiments. **c, d** Second experiment. The western blots in **a** and **c** were quantitated using ImageJ software and are shown in **b** and **d** respectively.

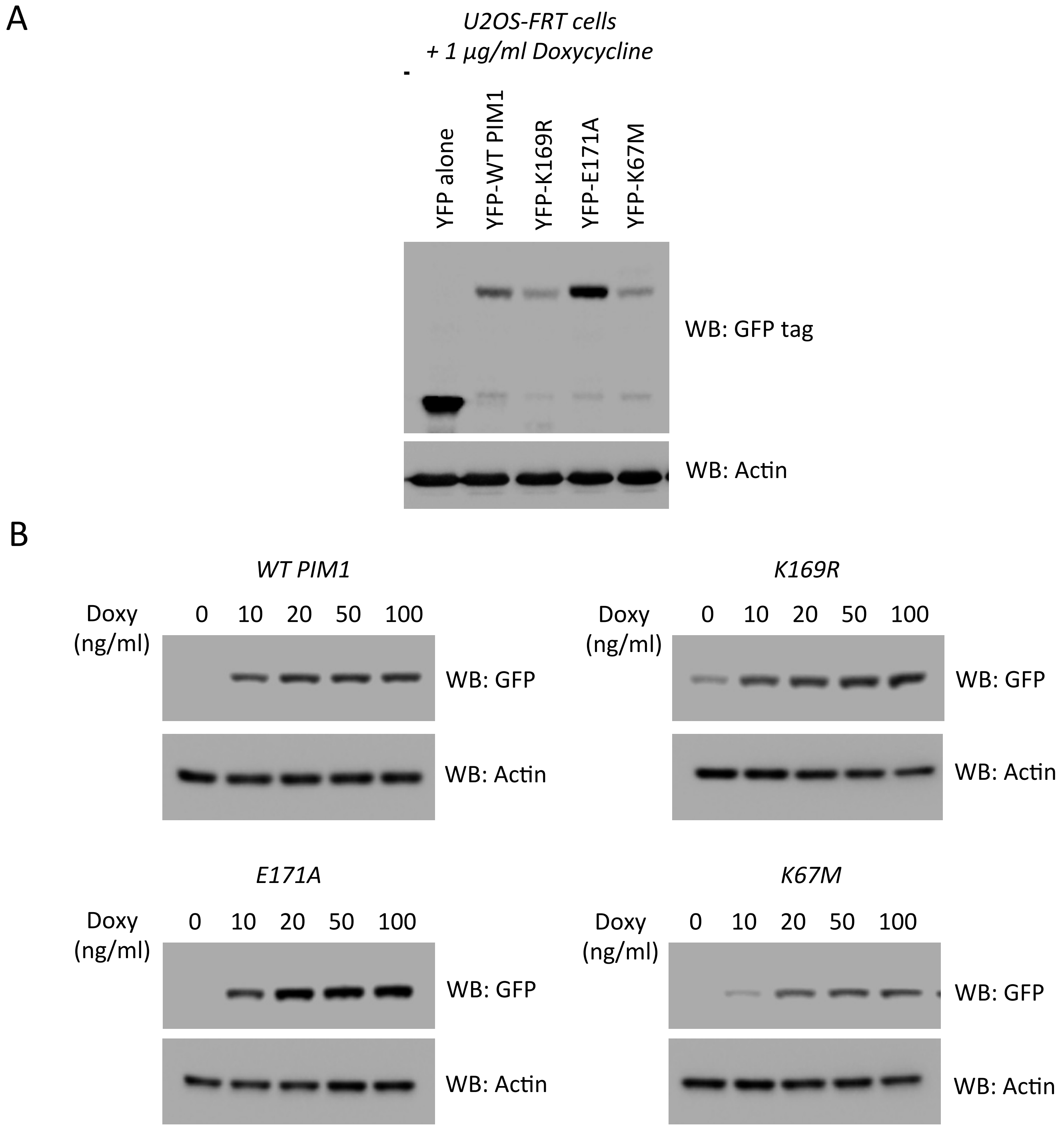
A
U2OS-FRT cells
+ 1 µg/ml Doxycycline
-
YFP alone
YFP-WT PIM1
YFP-K169R
YFP-E171A
YFP-K67M
WB: GFP tag
WB: Actin
B
WT PIM1
Doxy (ng/ml)
0 10 20 50 100
WB: GFP
WB: Actin
K169R
Doxy (ng/ml)
0 10 20 50 100
WB: GFP
WB: Actin
E171A
Doxy (ng/ml)
0 10 20 50 100
WB: GFP
WB: Actin
K67M
Doxy (ng/ml)
0 10 20 50 100
WB: GFP
WB: Actin

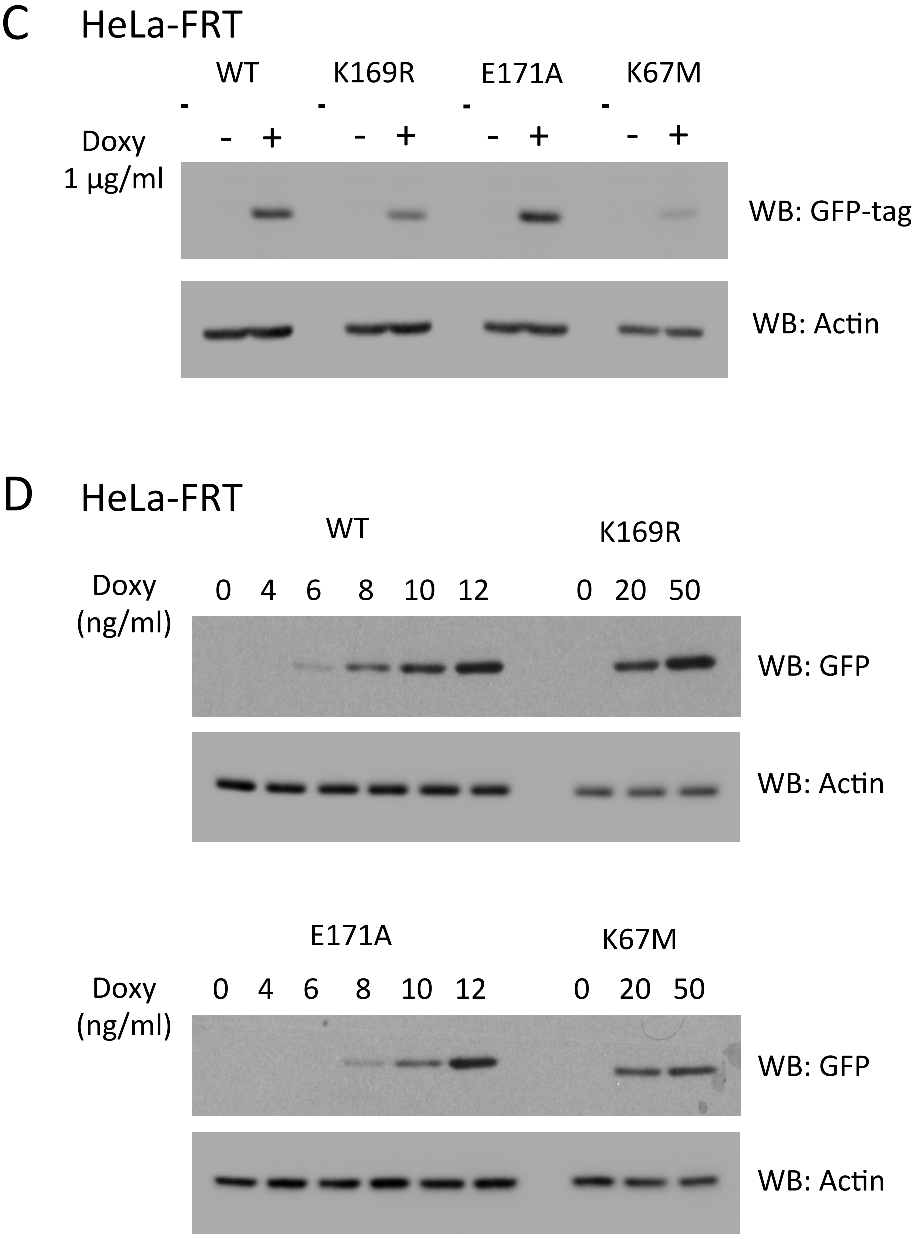


**Supplementary Figure 3.**

**Induction of PIM1 proteins in HeLa-FRT and U2OS-FRT cells following doxycycline addition.** The Flp-In TRex system (Thermo Fisher Scientific) was used to generate single-copy isogenic U2OS-FRT and HeLa-FRT derived lines that permitted inducible expression of the WT and mutant PIM1 proteins, as YFP-fusion proteins. **(A)** U2OS-FRT cells were treated with 1 µg/ml doxycycline for 24 hours, followed by western blotting using anti-GFP antibody. **(B)** U2OS-FRT cells expressing PIM1 were treated with increasing concentrations of doxycycline for 48 hours, followed by western blotting using anti-GFP antibody. **(C)** HeLa-FRT cells were assayed for the expression of YFP-PIM1 in the presence or absence of doxycycline (1 µg/ml) for 24 hours, followed by western blotting with GFP-tag antibody. **(D)** HeLa-FRT cells were treated with increasing concentrations of doxycycline for 48 hours, followed by western blotting with GFP-antibody.

A

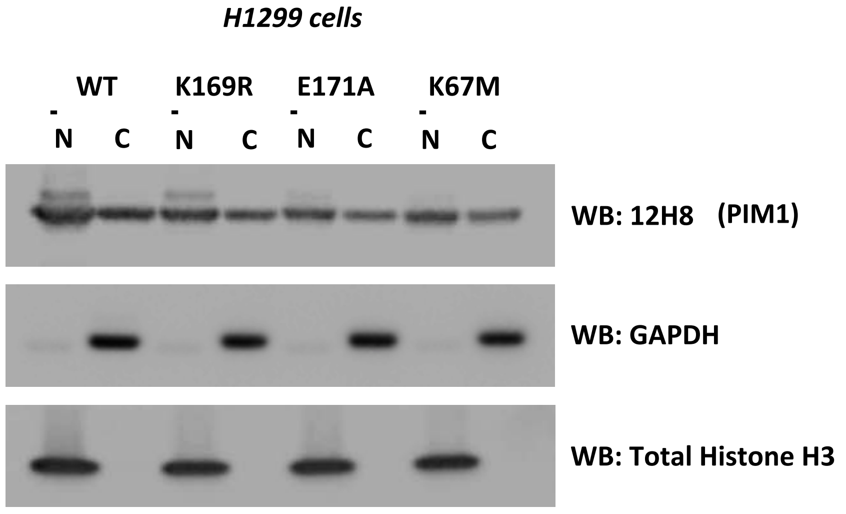


B

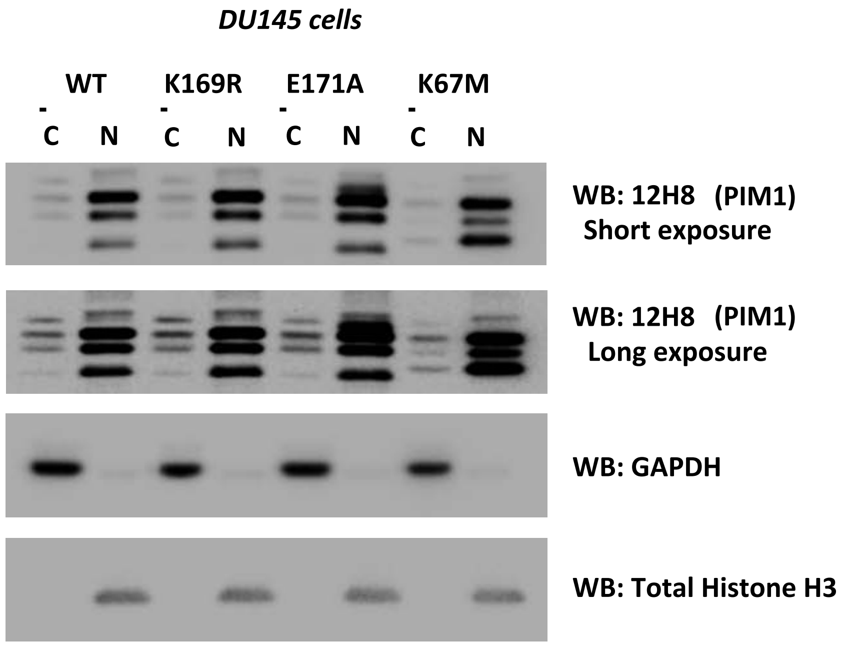


Iyer et al Sup Fig 4A, B

C

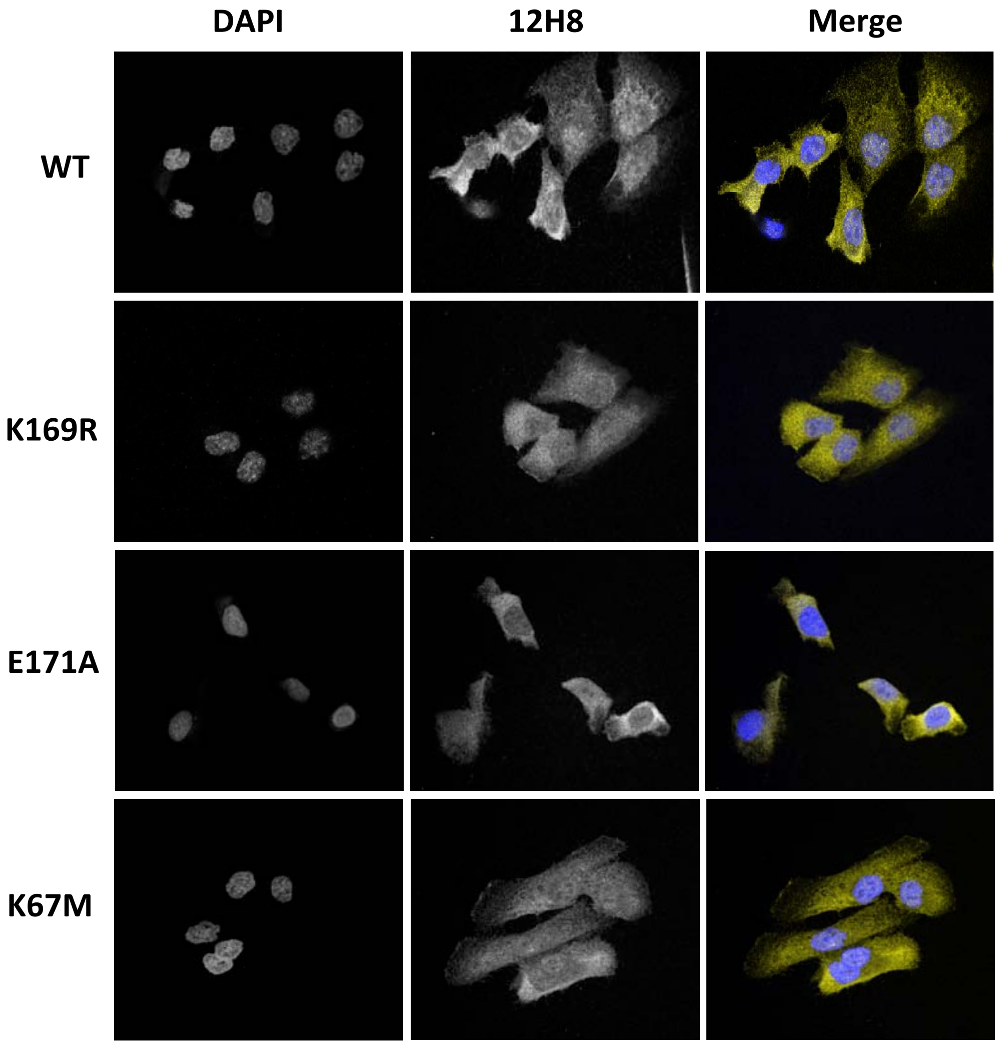


**Supplementary Figure 4.**

**Nuclear-cytoplasmic fractionation and cellular localisation of WT PIM1 and mutants**

**(A)** H1299 cells were transiently transfected with plasmid expressing myc-tagged WT PIM1 or mutants, and a nuclear-cytoplasmic fractionation was performed. Equal concentration of the nuclear and cytoplasmic fractions was resolved by SDS-PAGE and western blotting was performed using 12H8 (PIM1 antibody) **(B)** DU145 cells stably expressing different PIM1 proteins were treated with MG132 (20 µM for 6 hours) to equalise protein levels, and subjected to nuclear-cytoplasmic fractionation. Equal concentration of the nuclear and cytoplasmic fractions was resolved by SDS-PAGE and western blotting was performed using 12H8 (PIM1 antibody). In both experiments, GAPDH was used a cytoplasmic marker and Histone H3 as a nuclear marker to check efficiency of fractionation. **(C)** DU145 cells stably expressing WT PIM1 or mutants were grown on coverslips, and stained with 12H8 (PIM1) antibody (shown in yellow) overnight, followed by incubation with a fluorescent-labelled secondary antibody. DAPI was used to stain DNA or nuclear (blue). Images were acquired using a 63X oil immersion objective lens of a Leica SP5 confocal microscope. Representative images of different fields of view have been shown here.

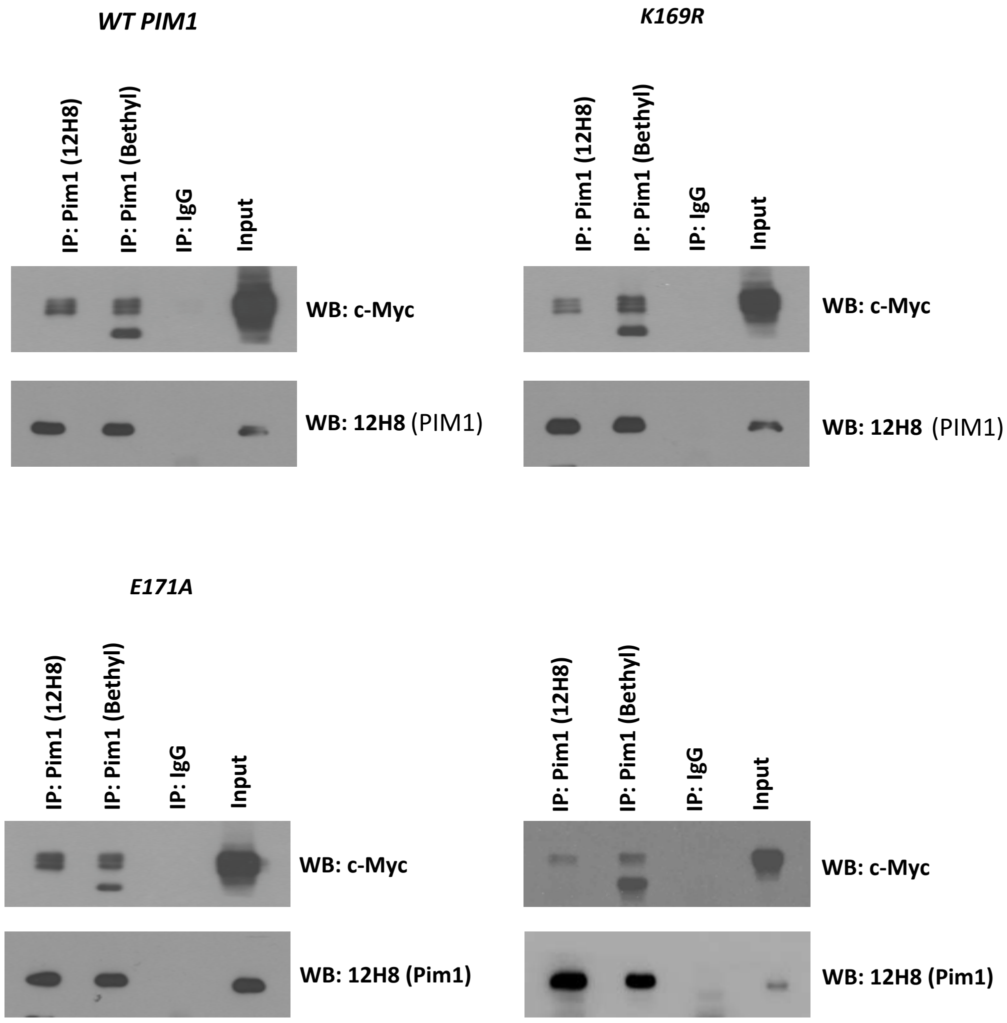


**Supplementary Figure 5.**

**Co-immunoprecipitation of PIM1 with its substrate, c-MYC**

H1299 cells were transfected with plasmids expressing c-MYC with either WT PIM1 or the mutants. Co-immunoprecipitation was performed using two PIM1 antibodies 12H8 and Bethyl or IgG negative control, and immune complexes were captured on Protein-A beads. Immunoprecipitated proteins were eluted by boiling the beads in 2X sample buffer, followed by western blotting for c-MYC or PIM1.

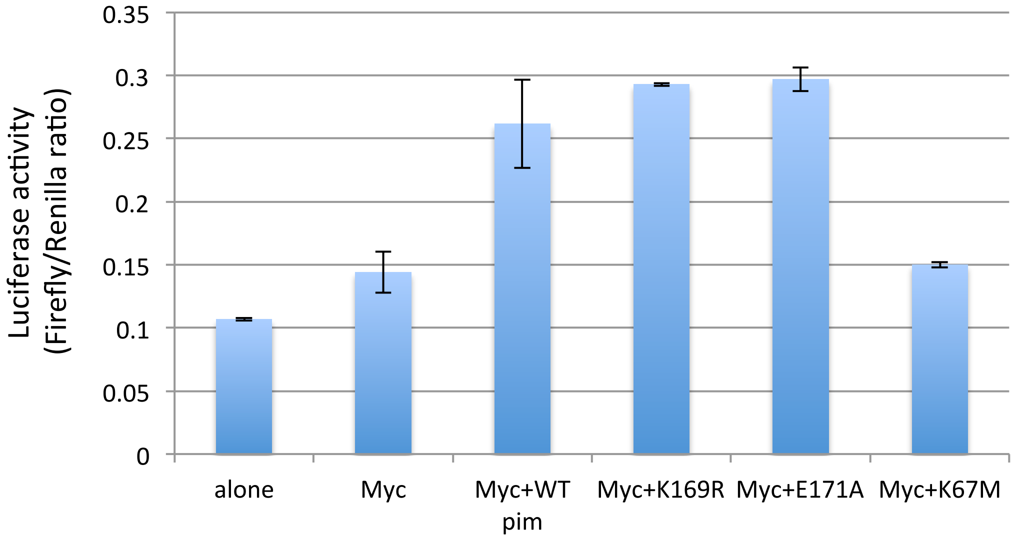


**Supplementary Figure 6.**

**Ability of of WT PIM1 and mutants to stimulate MYC-dependent transcription**

H1299 cells were transfected with the Myc responsive 4X E-box promoter/pGL3 vector with Myc alone or in combination with myc-tagged WT PIM1, K169R, E171A and K67M mutants respectively, and assayed for luciferase activity after 24 h.
